# Predictors of Delayed Antiretroviral Therapy Initiation, Mortality, and Loss to Followup in HIV Infected Patients Eligible for HIV Treatment: Data from an HIV Cohort Study in India

**DOI:** 10.1155/2013/849042

**Published:** 2013-10-29

**Authors:** Gerardo Alvarez-Uria, Raghavakalyan Pakam, Manoranjan Midde, Praveen Kumar Naik

**Affiliations:** Department of Infectious Diseases, Bathalapalli Rural Development Trust Hospital, Kadiri Road, Bathalapalli, Anantapur District, Andhra Pradesh 515661, India

## Abstract

Studies from Sub-Saharan Africa have shown that a substantial number of HIV patients eligible for antiretroviral therapy (ART) do not start treatment. However, data from other low- or middle-income countries are scarce. In this study, we describe the outcomes of 4105 HIV patients who became ART eligible from January 2007 to November 2011 in an HIV cohort study in India. After three years of ART eligibility, 78.4% started ART, 9.3% died before ART initiation, and 10.3% were lost to followup. Diagnosis of tuberculosis, being homeless, lower CD4 count, longer duration of pre-ART care, belonging to a disadvantaged community, being widowed, and not living near a town were associated with delayed ART initiation. Diagnosis of tuberculosis, being homeless, lower CD4 count, shorter duration of pre-ART care, belonging to a disadvantaged community, illiteracy, and age >45 years were associated with mortality. Being homeless, being single, not living near a town, having a CD4 count <150 cells/**μ**L, and shorter duration of pre-ART care were associated with loss to followup. These results highlight the need to improve the timely initiation of ART in HIV programmes in India, especially in ART eligible patients with tuberculosis, low CD4 counts, living in rural areas, or having a low socioeconomic status.

## 1. Introduction

In developed countries, people infected with HIV on antiretroviral therapy (ART) can have a similar life expectancy to the general population [1]. It is estimated that the implementation of ART programmes has added 15 million life years [2]. Nevertheless, 1.7 million people died of HIV-related pathologies in 2011, and more than 90% of them were living in low-income or middle-income countries [2].

Despite the fact that ART is free in many low-income and middle-income countries, studies from Sub-Saharan Africa have shown that 25% of patients in need of HIV treatment do not start ART [3]. However, data from developing countries outside Sub-Saharan Africa are scarce. 

With 2.4 million HIV infected people, India bears the third largest burden of HIV in the world [4]. While it is estimated that two-thirds of HIV infected patients are eligible for ART at the moment of HIV diagnosis [5], only 604,987 (35.6%) of the 1.7 million people living with HIV who were registered in Government ART centres had started ART by December 2012 [6]. The aim of this study is to describe the initiation of ART among ART eligible patients in a large cohort study in Anantapur, India. In particular, we aimed to find predictors of delayed ART initiation, death, and loss to followup.

## 2. Methods

### 2.1. Setting

The study was performed in Anantapur, a district situated in the south border of Andhra Pradesh, India. In Anantapur, 72% of the population live in rural areas [7], and there is a high prevalence of HIV infection in antenatal clinics [8]. 

Rural Development Trust (RDT) is a nongovernmental organization that provides medical care to HIV infected people free of cost, including medicines, consultations, or hospital admission charges. The Vicente Ferrer HIV Cohort Study (VFHCS) is an open cohort study that collects routine clinical data of all HIV infected patients who have attended RDT hospitals since June 2006. The cohort is fairly representative of the HIV population in the district, as it covers approximately 70% of all HIV infected patients registered in the district [9]. The HIV epidemic in the area is predominately driven by heterosexual transmission and it is characterized by poor socioeconomic conditions and high levels of illiteracy in the HIV population [10]. 

For this study, we selected HIV infected adults (>15 years) living in Anantapur and diagnosed with HIV between January 1, 2007, and November 4, 2011, and who became eligible for ART during this period. The selection of patients from the database was performed on September 14, 2012. During this period, ART was available free of cost and the CD4 cell count threshold for initiating ART was 250 cells/*µ*L [11–13]. According to Indian guidelines, CD4 count determinations were performed every six months [14]. Patients lost to followup (LTFU) were routinely searched for by phone calls and home visits by outreach workers, and in those patients who had died, relatives were asked about the date of death of the patient.

### 2.2. Variables

Age was defined as the period of time between birth and ART eligibility. 

Designation of the community of patients was performed by self-identification. Scheduled caste community is marginalised in the traditional Hindu caste hierarchy and, therefore, suffers social and economic exclusion and disadvantage. Scheduled tribe community is generally geographically isolated with limited economic and social contact with the rest of the population. Scheduled castes and scheduled tribes were considered socially disadvantaged communities.

Illiteracy was defined as not being able to read or write.

Patients were considered as living near an ART centre if they lived in a Mandal (administrative subdivision of districts in Andhra Pradesh; e.g., Anantapur district has 64 Mandals) with an ART centre or lived next to a Mandal with an ART centre. 

Patients were considered as living near a town when they lived in a Mandal containing a town with a population >100,000 people. Towns have better communications than rural areas. 

Poverty was defined as living with less than 1000 Indian rupees per month (approximately 18 US dollars in April 2013). 

Tuberculosis was defined as being diagnosed with tuberculosis within three months of becoming eligible for ART.

To calculate the duration of pre-ART care, we used the time period between the date of the first CD4 count determination (a marker of entry into HIV care) and the date of becoming ART eligible. 

### 2.3. Statistical Analysis

Statistical analysis was performed using Stata Statistical Software (Stata Corporation: Release 11, College Station, TX, USA). Missing values were imputed using multiple imputations by chained equation assuming missing at random [15]. The variables that were imputed were poverty (86 missing values), homelessness (123 missing values), illiteracy (4 missing values), and marital status (11 missing values). Time was measured from the date of ART eligibility to the date of ART initiation, death, or the last visit, whatever came first. Patients who did not initiate ART and who did not come to the clinics within six months before the end of the study period were considered LTFU. Kaplan-Meier estimations of the cumulative incidence and Cox regression multivariable analysis assume that the distribution of censoring times and the time to event distribution are independent of each other [16]. When studying the cumulative incidence of ART initiation, a group of patients will be censored at death or, in cases of LTFU, at the last visit date. However, dead patients will not be able to start ART and patients LTFU have a higher risk of late ART initiation and death [17]. Including these patients in standard survival models may lead to an overestimation of the event of interest, in this case the cumulative incidence of ART initiation. Thus, multivariate analysis and estimation of the cumulative incidence of ART initiation were performed using competing risk proportional hazard models with death before ART initiation and loss to followup as competing events [18]. These models estimate subdistribution hazard ratios (SHRs), which can be interpreted similarly to hazard ratios estimated by Cox proportional models, but they take into account the hazard of the competing events [16]. To avoid collinearity and convergence problems in multivariate models, the group with the largest sample size was selected when variables had more than two groups [19]. Cumulative incidence of ART initiation, loss to followup, and death was estimated using the “stcompet” command in Stata [20, 21]. The proportional hazard assumption was assessed performing log-log survival curves based on Schoenfeld residuals [22]. The study was approved by the Ethical Committee of the RDT Institutional Review Board.

## 3. Results

We identified 4105 patients who were eligible for ART during the study period.  Table 1 shows the baseline characteristics of the patients. Forty percent were women and the median age was 34.1 years (interquartile range (IQR) 28–40). Twenty-seven percent belonged to a disadvantaged community, 58% were illiterate, 36% had a monthly income <1000 INR, and 9% were homeless. Nearly two-thirds were married, 32% lived near an ART centre, and 45% lived near a town. Sixteen percent of patients were diagnosed with tuberculosis within three months of becoming eligible for ART. The median CD4 count at ART eligibility was 128 cells/*µ*L (IQR 73–190), and only 15% of patients were in pre-ART care longer than three months. The median CD4 count at ART eligibility was 111 cells/*µ*L (IQR 62–176) in 2007, 123 cells/*µ*L (IQR 76–183) in 2008, 137 cells/*µ*L (IQR 80–202) in 2009, 139 cells/*µ*L (IQR 80–189) in 2010, and 123 cells/*µ*L (IQR 61–192) in 2011.

During the study period, 3205 patients started ART, 375 died before ART initiation, and 425 were LTFU. The study included 1468 person-years. The median time for ART initiation was 1.8 months (IQR 1.5–2.7), and the median time to death was 2.8 months (IQR 1.6–8.9). Among patients who were LTFU, the median follow-up time was 0.9 months (IQR 0–5). 

**Figure 1:**
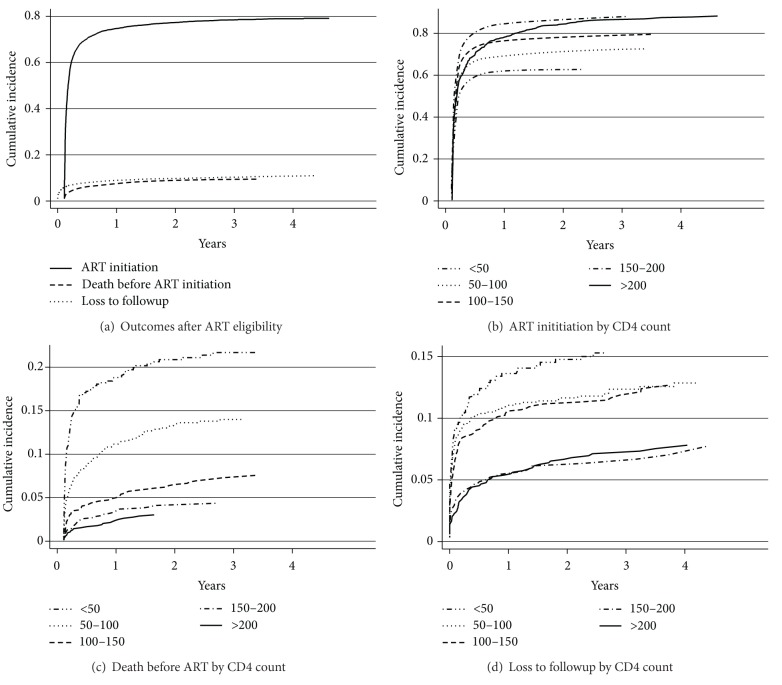
Cumulative incidence of antiretroviral therapy (ART) initiation, death before ART initiation, and loss to followup since ART eligibility (a), ART initiation by CD4+ lymphocyte count (b), mortality by CD4 overall and stratified by CD4+ lymphocyte count (c), and loss to followup by CD4+ lymphocyte count (d).

**Figure 2:**
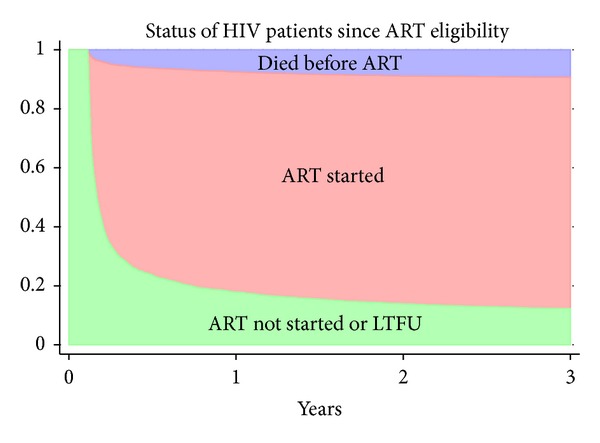
Cumulative incidence of mortality and antiretroviral therapy (ART) initiation in 4105 patients eligible for ART in Anantapur, India. ART: antiretroviral therapy; LTFU: lost to followup.

Figure 1 shows the cumulative incidence of ART initiation, death, and loss to followup overall and stratified by CD4 counts. The proportion of patients starting ART at 3, 6, 12, and 36 months was 61.5% (95% CI, 60–62.9), 70.3% (95% CI, 68.8–71.6), 74.7% (95% CI, 73.3–76), and 78.4% (95% CI, 77.1–79.6), respectively. The proportion of patients who died before ART initiation at 3, 6, 12, and 36 months was 4.8% (95% CI, 4.2–5.5), 6.2% (95% CI, 5.5–7), 7.5% (95% CI, 6.7–8.3), and 9.3% (95% CI, 8.5–10.3), respectively. The proportion of patients LTFU at 3, 6, 12, and 36 months was 7% (95% CI, 6.2–7.8), 7.9% (95% CI, 7.1–8.8), 9% (95% CI, 8.1–9.9), and 10.3% (95% CI, 9.4–11.3), respectively. Compared with patients having CD4 counts 150–200 cells/*µ*L, those with CD4 counts >200 cells/*µ*L had a slower rate of ART initiation (Figure 1(b)). In Figure 1(c), we can observe that the proportion of patients who died was inversely proportional to the CD4 count. In Figure 1(d), the proportion of patients LTFU was higher in patients with CD4 counts <50 cells/*µ*L and in those with CD4 counts 50–150 cells/*µ*L.  Figure 2 shows a stacked graph of the status of HIV patients since ART eligibility.

**Figure 3:**
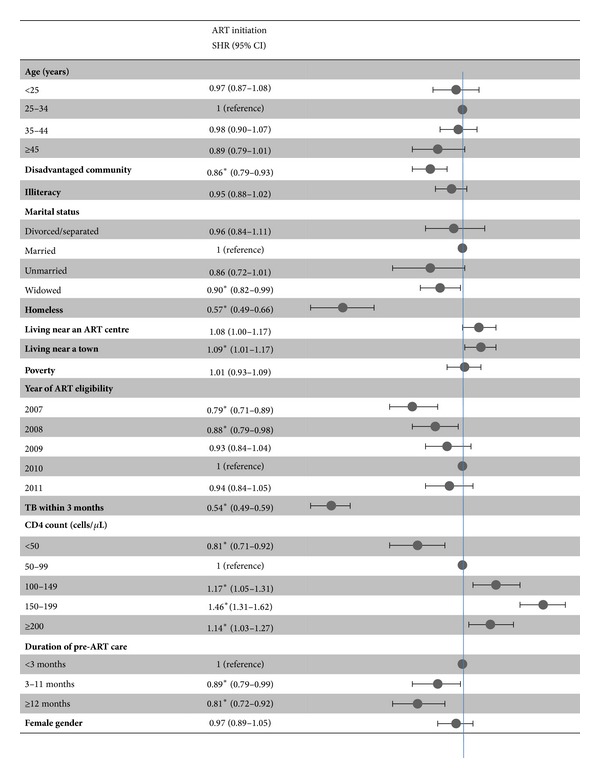
Factors associated with antiretroviral therapy initiation. ART: antiretroviral therapy; SHR: subdistribution hazard ratio; TB: tuberculosis. *P  value < 0.05.

Figure 3 shows the multivariate analysis of factors associated with ART initiation accounting for death and loss to followup as competing risks. Factors associated with a lower probability of ART initiation were diagnosis of tuberculosis within three months of ART eligibility, being homeless, lower CD4 count, longer duration of pre-ART care, belonging to a disadvantaged community, being widowed, not living near a town, and becoming eligible in 2007 or 2008.

**Figure 4:**
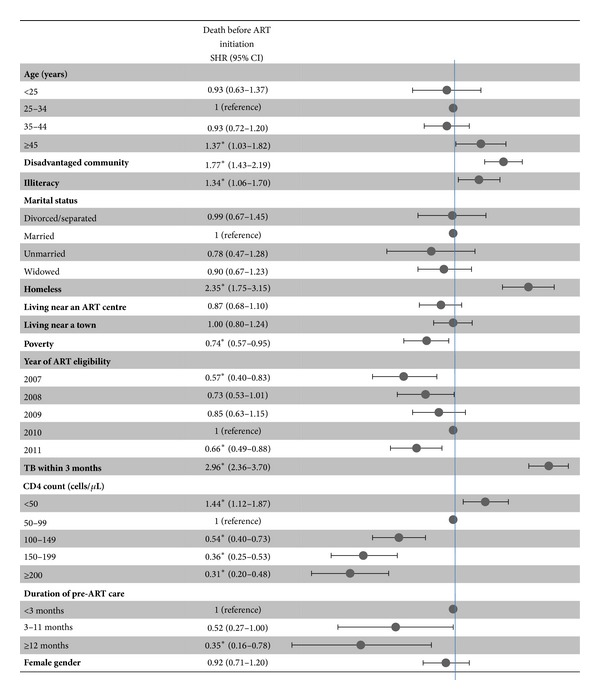
Factors associated with death before antiretroviral therapy initiation. ART: antiretroviral therapy; SHR: subdistribution hazard ratio; TB: tuberculosis. *P-value < 0.05.

Figure 4 shows the multivariate analysis of factors associated with mortality before starting ART accounting for ART initiation and loss to followup as competing risks. Factors associated with a higher probability of death were diagnosis of tuberculosis within three months of ART eligibility, being homeless, lower CD4 count, shorter duration of pre-ART care, belonging to a disadvantaged community, illiteracy, and age >45 years.

**Figure 5:**
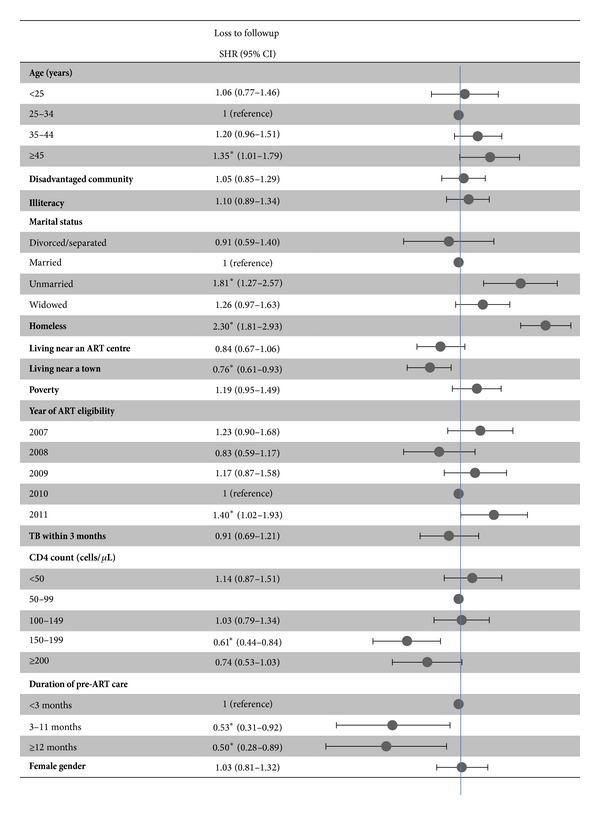
Factors associated with loss to followup before antiretroviral therapy initiation. ART: antiretroviral therapy; SHR: subdistribution hazard ratio; TB: tuberculosis. *P  value < 0.05.

Figure 5 shows the multivariate analysis of factors associated with loss to followup accounting for ART initiation and death as competing risks. Factors associated with a higher probability of loss to followup were being homeless, being unmarried, not living near a town, becoming eligible in 2011, CD4 counts <150 cells/*µ*L, and shorter duration of pre-ART care.

## 4. Discussion

The study describes the ART initiation and the attrition before starting ART of 4105 patients eligible for ART in a cohort study in Anantapur, India. After three years of followup, 9.3% of ART eligible patients died before ART initiation and 10.3% were LTFU. In a meta-analysis of the pre-ART attrition in Sub-Saharan Africa [3], it was estimated that 10.8% (95% CI, 4.6–17) of ART eligible patients die before ART initiation and 13.2% (95% CI, 9.3–17.1) are LTFU. These data indicate comparable rates of attrition of ART eligible patients in Sub-Saharan Africa and India, highlighting the need to improve the timely ART initiation in resource-limited settings.

In the present study, the median CD4 count at ART eligibility was low (128 cells/*µ*L) and did not change substantially during the five years of the study. In accordance with Sub-Saharan African studies [16, 23–26], patients with lower CD4 counts were less likely to initiate ART and had a higher risk of death and loss to followup. Typically, preparation of ART requires two or three counselling sessions during 4–6 weeks before ART is started. However, we found that patients with CD4 counts <150 cells/*µ*L had a high risk of death and loss to followup, so these patients may benefit from “fast tracking” interventions to rule out tuberculosis and other opportunistic infections and to provide intensive adherence counselling [27]. The use of mobile phone text messaging, phone calls, or home visits could potentially reduce the time period from the collection of blood for CD4 count enumeration to the moment the patient is informed about the need for ART initiation [25, 28, 29]. Interestingly, the rate of ART initiation in patients with CD4 counts >200 cells/*µ*L was lower than in patients having CD4 counts 150–200 cells/*µ*L, which may be related to the fact that those with higher CD4 counts were more likely to be asymptomatic and might have been more reluctant to start ART [30].

To our knowledge, this is one of the first studies to describe predictors of delayed ART initiation of ART eligible patients in India. The study describes a “real life” situation, so these results can be generalized to other similar sites in India. In general, factors related to having a low socioeconomic status were associated with poorer outcomes. Being homeless was strongly associated with delayed ART initiation and a higher risk of death and loss to followup. Belonging to a disadvantaged community was associated with delayed ART initiation and a higher mortality. Illiteracy was also associated with a higher mortality. Not living near a town was associated with delayed ART initiation and a higher risk of loss to followup. Previous studies have shown that travel time and travel costs can be major impediments for HIV patients to come to the clinics [16, 17, 25, 31], supporting the current policy of decentralization of ART centres by the Government of India. These results indicate that patients with a low socioeconomic status or living in rural areas are at higher risk of attrition, even when ART is given free of cost, and may need economical support to overcome the travel costs from their homes to the ART centres. 

Sixteen percent of the patients were diagnosed with tuberculosis within three months of ART eligibility, and these patients had a lower probability of ART initiation and a higher risk of death. This is in line with the high mortality observed in HIV patients with tuberculosis in a previous study from our cohort [32]. In Soweto, South Africa [26], patients with tuberculosis were also more likely to initiate ART late. This may be explained by patients being reluctant to take both ART and antituberculous treatment and concerns of healthcare workers about the tuberculosis immune reconstitution inflammatory syndrome related to early initiation of ART [27]. However, there is increasing evidence indicating that early initiation of ART reduces mortality in HIV patients with tuberculosis [27].

Single patients were more likely to be LTFU and widowed patients were more likely to delay ART initiation. In Soweto, South Africa [26], patients who were single, divorced, or widowed were more likely to refuse ART, and the main reason given for the refusal was that they were “feeling healthy.” In Durban, South Africa [23], patients having an HIV infected relative or friend were more likely to start ART. These findings suggest that ART eligible patients should be encouraged to disclose their HIV status to relatives or friends in order to obtain social support to initiate ART [33, 34]. 

In contrast with a study from Malawi [35], longer duration of pre-ART care was associated with delayed ART initiation. This difference might be explained by the fact that, in our study, patients were considered eligible at the time of performing the CD4 count, whereas in the Malawian study patients were considered eligible when informed of their CD4 count result. It is possible that, in our study, patients already in pre-ART care were informed of the need for ART initiation at the next visit to the clinics after a routine collection of blood for CD4 count enumeration, which typically happens after 3–6 months, whereas patients who were recently diagnosed with HIV were told to come back to the clinics in a few days to be informed of their CD4 count result. Nevertheless, patients with longer periods of pre-ART care had a lower mortality and a lower risk of loss to followup. Patients with longer periods of pre-ART followup have more time to overcome the initial psychological, sociocultural, and operational barriers of patients recently diagnosed with HIV (i.e., acceptance of the HIV diagnosis, disclosure to family and friends, trust in counselors, and health workers of HIV centres) and have received more counselling sessions than patients found to be eligible at the first CD4 count determination. 

The study has some limitations. Loss to followup was considered as a competing risk for death and ART initiation, but patients LTFU may not be lost forever, as they may reengage in the future or enrol in other ART centres. However, patients LTFU are more likely to initiate ART late or may die before attending other healthcare facilities [17, 23, 25, 31, 36, 37], so censoring patients LTFU at the last visit would have created a significant bias in the study [17]. Moreover, including loss to followup as a competing risk allowed for studying risk factors associated with loss to followup accounting for ART initiation and death.

## 5. Conclusion

This is one of the first studies to describe the outcomes of ART eligible patients in a large cohort from a resource-limited setting outside Sub-Saharan Africa. We found that approximately 20% of ART eligible patients die or are LTFU, and this percentage is higher in patients with lower CD4 counts. Factors related to having a poor socioeconomic status, tuberculosis, and shorter duration of pre-ART care were associated with a higher risk of death and loss to followup. These findings could be used to increase the number of patients receiving ART in India, by implementing targeted intervention for those patients at a higher risk of programme attrition.

## Figures and Tables

**Table 1:** Baseline characteristics of patients eligible for antiretroviral therapy initiation.

	*N* (%)
Age (years)	
<25	564 (13.74)
25–34	1822 (44.38)
35–44	1150 (28.01)
≥45	569 (13.86)
Disadvantaged community	1113 (27.11)
Illiteracy	2390 (58.28)
Marital status	
Divorced/separated	250 (6.11)
Married	2821 (68.91)
Unmarried	238 (5.81)
Widowed	785 (19.17)
Homeless	370 (9.29)
Living near an ART centre	1304 (31.77)
Living near a town	1756 (42.78)
Poverty	1461 (36.35)
Year of ART eligibility	
2007	633 (15.42)
2008	692 (16.86)
2009	899 (21.9)
2010	1067 (25.99)
2011	814 (19.83)
TB within 3 months	648 (15.79)
CD4 count (cells/*µ*L)	
<50	621 (15.13)
50–99	915 (22.29)
100–149	889 (21.66)
150–199	843 (20.54)
≥200	837 (20.39)
Duration of pre-ART care	
<3 months	3491 (85.04)
3–11 months	293 (7.14)
≥12 months	321 (7.82)
Female gender	1635 (39.83)

ART: antiretroviral therapy; TB: tuberculosis.
